# Diagnostic validity of early proximal caries detection using near-infrared imaging technology on 3D range data of posterior teeth

**DOI:** 10.1007/s00784-021-04032-1

**Published:** 2021-10-12

**Authors:** Friederike Litzenburger, Katrin Heck, Dalia Kaisarly, Karl-Heinz Kunzelmann

**Affiliations:** Department of Conservative Dentistry and Periodontology, University Hospital, LMU Munich, Goethestr. 70, 80336 Munich, Germany

**Keywords:** X-ray microtomography, Sensitivity and specificity, Reproducibility of results, Dental caries, Near-infrared imaging, Near-infrared reflection

## Abstract

**Objectives:**

This in vitro study analysed potential of early proximal caries detection using 3D range data of teeth consisting of near-infrared reflection images at 850 nm (NIRR).

**Materials and methods:**

Two hundred fifty healthy and carious permanent human teeth were arranged pairwise, examined with bitewing radiography (BWR) and NIRR and validated with micro-computed tomography. NIRR findings were evaluated from buccal, lingual and occlusal (trilateral) views according to yes/no decisions about presence of caries. Reliability assessments included kappa statistics and revealed high agreement for both methods. Statistical analysis included cross tabulation and calculation of sensitivity, specificity and AUC.

**Results:**

Underestimation of caries was 24.8% for NIRR and 26.4% for BWR. Overestimation was 10.4% for occlusal NIRR and 0% for BWR. Trilateral NIRR had overall accuracy of 64.8%, overestimation of 15.6% and underestimation of 19.6%. NIRR and BWR showed high specificity and low sensitivity for proximal caries detection.

**Conclusions:**

NIRR achieved diagnostic results comparable to BWR. Trilateral NIRR assessments overestimated presence of proximal caries, revealing stronger sensitivity for initial caries detection than BWR.

**Clinical relevance:**

NIRR provided valid complement to BWR as diagnostic instrument. Investigation from multiple angles did not substantially improve proximal caries detection with NIRR.

## Introduction

The number of diagnostic methods available to dentists for caries detection has multiplied in recent years. Due to new preventive and microinvasive therapy strategies, there is an increasing need to be able to detect and correctly assess caries at an early stage [1, 2]. With the established procedures of visual inspection and bitewing radiography, both healthy tooth structure and advanced cavitated lesions can be correctly identified [3–5]. However, early proximal caries is not detected adequately [6]. In the context of new therapeutic approaches, high sensitivity for early caries detection is desirable, necessitating other diagnostic methods for the detection and assessment of initial lesions with high accuracy.

Over the last three decades, numerous techniques have been developed and investigated to meet this challenge. Most techniques, such as quantitative light-induced fluorescence, laser fluorescence, electrical conductance, impedance spectroscopy and photothermal radiometry, are well suited for the assessment of smooth surfaces [7]. Lesions in the proximal region can be visualized by transillumination with visible light or optical coherent tomography (OCT), although OCT devices are currently so expensive that there will be no system available for general dental practice under economic conditions in the foreseeable future. Transillumination with near-infrared (NIR) light is expected to make approximal caries visible and has been protected by a patent for wavelengths above 795 nm [8]. Theoretically, it should also be possible to visualize caries by reflection of NIR light. There are already commercially available devices for this method, e.g., VistaCam (Dürr Dental, Bietigheim-Bissingen, Germany) or the iTero Element 5D scanner (Align, San José, CA, USA). VistaCam (Dürr Dental, Bietigheim, Bissingen) uses two light-emitting diodes (LEDs) at 850 nm for the detection of proximal caries of permanent molars and premolars. Lederer et al. have shown that near-infrared reflection (NIRR), as applied in the form of the VistaCam system, has a weaker diagnostic performance than digital bitewing radiography (BWR), as its sensitivity values for the detection of enamel lesions did not even reach half the performance of radiography [9].

**Fig. 1:**
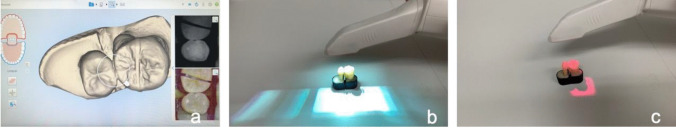
The application of the three-dimensional near-infrared reflection scanner is visualized by a monitor with the appropriate software (***a***). The tooth is illuminated either with a white LED (***b***) or a red laser (***c***)

The iTero Element 5D scanner, which was launched in the dental market in 2019, is an alternative to the VistaCam system. The scanner allows three-dimensional (3D) data of the dentition to be collected while simultaneously taking images of the teeth with nearly confocal imaging. An additional integrated NIR LED, which emits light at 850 nm, enables the detection of proximal caries lesions by NIRR. Compared to a Class 1 red laser at 680 nm and a white LED at 530–600 nm, which are also incorporated into the scanner, only the NIR light source has the potential to increase the light optical diagnostic performance because of the different properties of light scattering of sound and carious enamel at wavelengths around 800 nm (Fig. 1) [8].

The combination of a 3D scanner and diagnostic function with confocal illumination is an innovation. The exact position of the two-dimensional (2D) images relative to the 3D dataset using 3D range data makes it possible to project the 2D data onto the 3D surface reconstruction to perform so-called texture mapping [10]. Since images are taken from different angles, the tooth surface can be recorded from all sides. The area of interest can then be viewed on the monitor from all directions in an easy-to-read display. However, the 2D projection is only the first step in the sense of a proof of concept. The data have the potential to enable true 3D localization of carious defects in the sense of optical tomography [11–13].

The aim of this study was to compare the diagnostic performance of the iTero Element 5D scanner for the detection of early proximal caries with that of BWR. Micro-computed tomography (µCT) was used as a reference to estimate the diagnostic potential of this scanner.

A hypothesis was formulated that the diagnostic performance of the 3D intraoral scanner with NIRR at 850 nm as an additional diagnostic function would be comparable to that of BWR for the detection of early proximal caries [9].

## Methods and materials

### Tooth selection and sample preparation

The sensitivity for the detection of enamel caries with NIRR is assumed to be 15% [9]. This method aimed to increase sensitivity to 30%, with a power of over 90%, an alpha of less than 0.05 and a caries prevalence of 50% [14]. These assumptions required a minimum number of 250 samples. Two hundred fifty extracted permanent molars and premolars were selected from a pool of extracted teeth of anonymous patients. The experimental procedures were approved by the Ethics Committee of the Medical Faculty, Ludwig Maximilians University in Munich, Germany (488–15 UE).

**Fig. 2:**
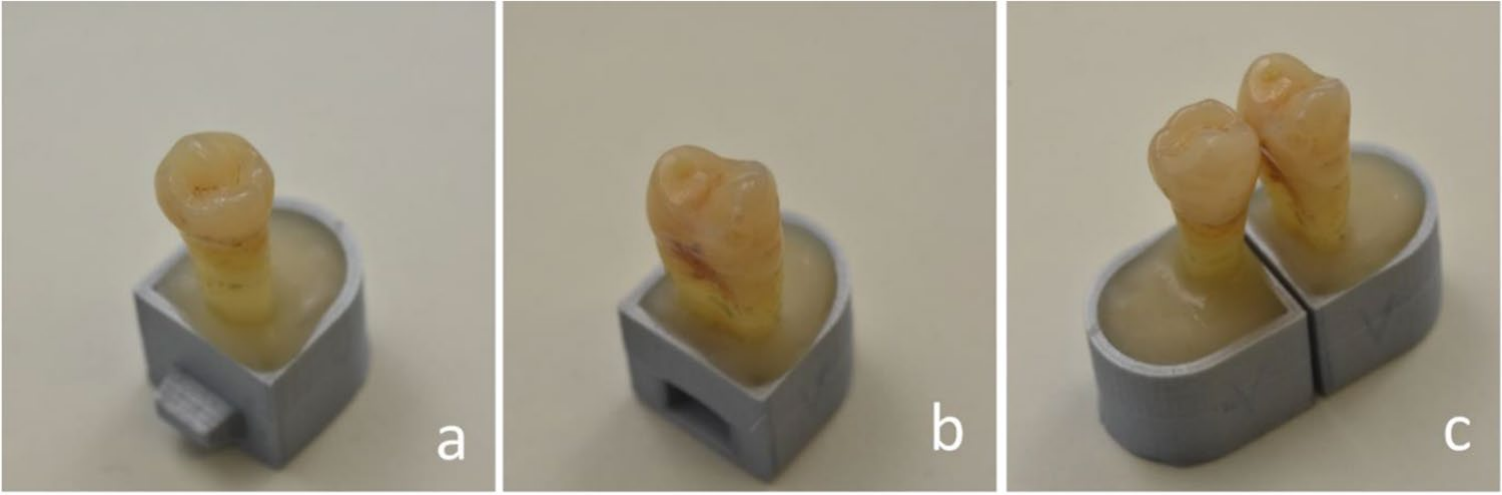
The teeth were fixed with composite material in three-dimensionally printed specimen holders. (**a**) Male holder, (**b**) female holder and (**c**) two specimens connected by a magnetic female-male key-lock joint

The teeth were visually examined according to the International Caries Detection and Assessment System II (ICDAS) without simulation of a proximal contact area between adjacent teeth [15]. To meet the inclusion criteria, all samples were free of any kind of restoration and of clearly identifiable structural changes or damages other than proximal carious lesions. One proximal surface of each tooth was selected for the study with the goal of obtaining a nearly even distribution of healthy (n = 131) and carious proximal surfaces (n = 119) according to the assumed prevalence of 50%. Of the carious surfaces, 112 were affected by enamel caries (ICDAS 1–3), and 7 were affected by dentin caries (ICDAS 4–5). The samples were cleaned of any residues using manual scalers and assigned a unique identification number (ID). The samples were randomly arranged in 125 pairs with each pair containing odd and even IDs. This allocation was performed in MS Excel (Microsoft Excel 2016, Redmond, WA, USA) with the “random numbers” formula. A sample holder specially designed for the study requirements was printed 250 times in 3D [14]. The teeth were vertically mounted, and the roots were fixed with the lower half in composite (Luxatemp Star, DMG, Hamburg, Germany) in the sample holder. Using a lock-and-key fixation method, the pairs were arranged to mimic the natural proximal contact area as closely as possible (Fig. 2). This connection was reproducibly fixable via a magnet that was polymerized at the bottom of the container. The ID was milled into the back of each sample holder. All samples and their holders were stored in Ringer’s solution at 4 °C between measurements.

### Three-dimensional near-infrared reflection

The magnetically coupled sample pairs were fixed on a metal plate and then scanned with the iTero Element 5D scanner with NIRR mode activated.

**Fig. 3:**
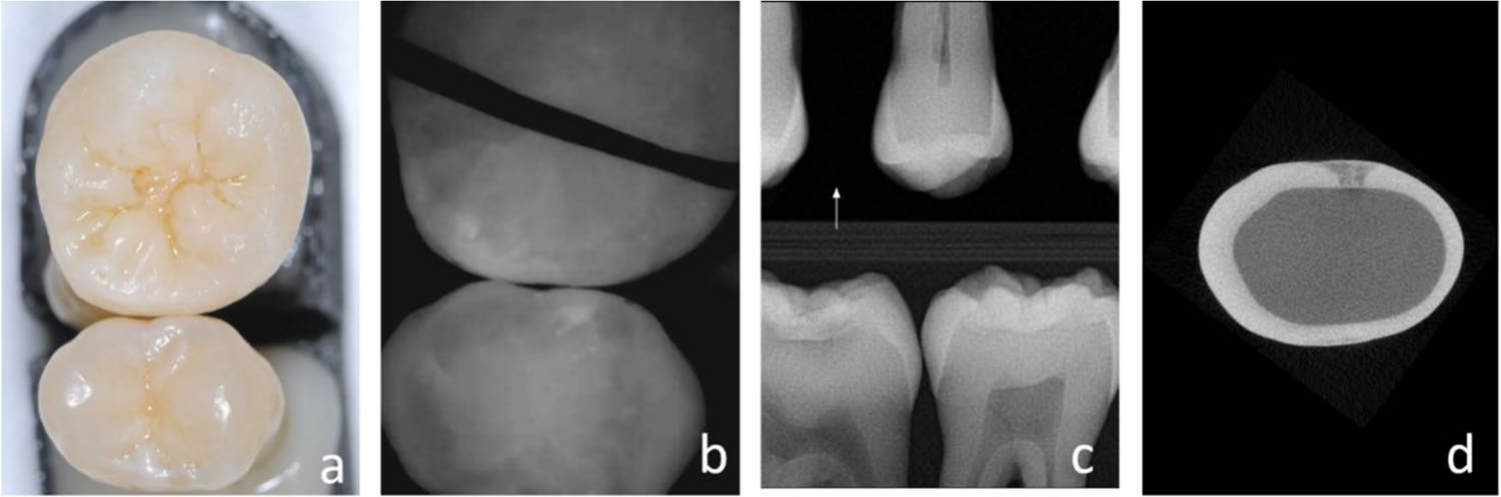
A non-cavitated caries lesion in a premolar that is visually undetectable (***a***). The caries lesion is visible with NIRR (white spot) and the black line marks the tooth that is not in the region of interest (***b***). The lesion was undetectable using X-rays and the arrow marks the side of interest (***c***). Micro-computed tomography reveals the presence of an initial dentin lesion (***d***)

The scanner software automatically projected the NIRR images onto the 3D range dataset (Fig. 1). The 3D dataset could be rotated as desired for visual assessment using the software, but this introduced a problem in that it was easy to inadvertently diagnose the wrong tooth when teeth within a tooth pair looked similar. To avoid this problem, which is unique to our experimental samples, the tooth that was not diagnosed in each case was marked using a thin black paper strip (Fig. 3b). The free rotation of the data sets could have negatively affected the test–retest variability, as well as the interrater reliability tests. For this reason, these tests were performed using screenshots. The 3D datasets populated with NIRR images were interactively aligned on the monitor so that one setting each allowed viewing of the proximal contact from occlusal, buccal, or lingual views. The views adjusted in this manner were exported as portable network graphics files for further evaluation and saved with the corresponding IDs. The evaluation was performed in a darkened room (blinds were 2/3 closed, windows facing north) on a monitor calibrated using the test pattern for the daytime constancy test according to DIN 6868–157 [16].

### Digital bitewing radiography

The exact procedure of bitewing radiograph acquisition with the help of an X-ray phantom was recently published in detail [14]. This phantom enables radiography of the relevant surfaces with and without direct contact with an adjacent tooth. It also takes into account antagonistic and adjacent teeth to minimize the influence of the automatic exposure setting and to ensure a clinically relevant overall appearance of the radiographs. To avoid hindering the evaluation of the radiographs by overlapping in the area of the proximal contacts and to enable the best possible radiographic diagnosis, the tooth pairs were radiographed without proximal contact for this study. All radiographs were taken using a Heliodent DS Dental X-ray unit (Sirona, Bensheim, Germany, 60 kV, 7 mA, 200 mm FHA cone, 0.08 s) and a digital charged-coupled device (CCD) sensor (Intra-Oral II CCD sensor, Sirona, Bensheim, Germany, sensor size 30.93 × 40.96 × 7.0 mm).

### Micro-computed tomography

Each sample embedded in its containers was mounted vertically in a 16.5-mm-diameter, cylindrical, water-filled plastic container and assessed with a µCT40 micro-computed tomography scanner (Scanco Medical, Bassersdorf, Switzerland) at 70 kV and 114 μA with a 16.5 mm field of view. The scan resolution was 512 × 512 points with a pixel size of 0.032 mm. After reconstructing the raw data (RSQ files) into 3D datasets (ISQ files), Fiji, a distribution of ImageJ, was used for further image processing [17].

### Calibration and training for the evaluation of findings of all index test methods and the reference test method

Calibration and training of the two examiners (K.H. and F.L.) were performed separately for the evaluation of NIRR, BWR and µCT findings by an experienced trainer (K.H.K.) and was subdivided into three parts. The first part consisted of theoretical training in which the trainer provided information about the diagnostic procedures and their classifications. The different configurations of NIRR findings that were new to all investigators of the group were analysed and discussed in the group so that a standardized evaluation process could be defined. The following appointment was a training section in which different findings of all four methods were evaluated and discussed. The examiners evaluated different findings, while reasons for differences were analysed and a consensus was achieved. The third part was the phase in which inter- and intra-examiner reliability of calibration was determined according to a new set of samples. This resulted in an agreement of more than 90% for the findings of all test methods as well as the reference test.

### Evaluation of the findings of the index test methods and the reference test method

The evaluation of the image-based findings (NIRR, BWR and µCT) was performed by the calibrated examiners independently after at least 5 min of eye adaptation to the room environment. The evaluation took place in two cycles in random order with an interval of two weeks. At the end of each cycle, a consensus was reached for different ratings.

In NIRR images, carious lesions in the enamel appeared as light areas compared to sound enamel due to increased light scattering and were scored positively (Fig. 3). In principle, occlusal representation provides the opportunity to detect the transition between enamel and dentin, since dentin scatters NIR light more strongly than enamel. This differentiation was not possible for all specimens. For this reason, the frequency that the enamel-dentin junction (EDJ) was recognizable in the occlusal NIRR images was evaluated. A distinction between sound (0) and carious (1) areas was performed for all three views, occlusal, buccal and lingual, meaning three scorings per sample. For the trilateral assessment of the NIRR images, all three scorings of a proximal surface (buccal, occlusal or lingual) were combined. A surface was considered diseased if only one angle scored 1 and healthy if all three angles scored 0.

The BWR findings were scored according to Marthaler et al., in which the absence of radiolucency (0), the presence of radiolucency in the outer or inner half of the enamel (1, 2) and the outer or inner half of dentin (3, 4) were evaluated [18]. Unclear representations that were ambiguous or not readable were defined as not assessable (na) (5).

Both examiners evaluated the µCT data to quantify the reference test method. As previously reported, the evaluation of the 3D datasets has been improved by segmentation and automatic centreline determination for dentin and enamel [14]. To determine the point closest to the pulp of a proximal lesion, the datasets were scrolled through vertically until the corresponding horizontal slice of the image stack was found and counterchecked with the corresponding vertical section. The digitally computed centrelines in the enamel and dentin were used to score the lesions according to the absence of radiolucency (0), the presence of radiolucency in the outer (1) or inner half of the enamel (2) and the presence of radiolucency in the outer (3) or inner half of dentin (4).

### Statistics

The sample size calculation was conducted using the Proc Power procedure with SAS/STAT software (SAS/STAT, Version 15.1, Cary, NC, USA), and for further statistical analysis, SPSS software (IBM SPSS Statistics for Windows, Version 25.0, Armonk, NY, USA) was employed. The NIRR findings were evaluated as sound (0) and carious (1). The findings of BWR and µCT were also evaluated as either sound (0) or carious (1–4) accordingly. Calculation of sensitivity and specificity was performed, and receiver operating curves (ROCs) were generated. The NIRR findings were separated into two approaches of analysis: first, occlusal assessment of each relevant proximal surface was performed separately and second, trilateral assessment of the combination of occlusal, buccal and lingual sites of each proximal surface was performed. Multiple comparisons of AUCs within the thresholds were performed using easyROC and the Bonferroni method [19]. The interpretation of the AUC values was 0.5 = no discrimination; 0.5–0.7 = poor to fair discrimination; 0.7–0.8 = acceptable discrimination; 0.8–0.9 = excellent discrimination; and AUC ≥ 0.9 = outstanding discrimination [20]. Overall accuracy was calculated as the correctly classified sites divided by the total number of reference sites and expressed as a percentage. Likewise, overestimation was calculated as the proportion of false-positive (FP) values divided by the number of reference sites, while underestimation was calculated as the proportion of false-negative (FN) values divided by the number of reference sites. Reliability assessment was calculated using linear weighted Cohen’s kappa (wκ), where a 1-category difference could be considered less severe than a 2-category difference [21]. Weights ranged from 0 to 1, and the weight for cells where the examiners disagreed exactly equalled 1. For cells in the lower left or upper right corners with the largest disagreement, the weight equalled 0. Each weight (W) for any cell was calculated by the formula Wxy = 1-(|x–y|)/z, with x and y being the categories and z the total number of categories. A two-sided significance level was set at α = 0.05 for all tests. The overall accuracy was defined as the percentage of correctly classified diagnostic findings (both sound and carious) in relation to the total number of diagnostic findings.

## Results

Caries classification based on µCT imaging showed almost perfect agreement between the two investigators (linear wκ 0.99, confidence interval (CI): 0.97–1.00). Of the 250 samples, 63.2% (n = 158) were found to be sound, and 36.8% (n = 92) had caries in the proximal contact area (Table 1).

**Table 1:** Cross-table for the ratings of three-dimensional near-infrared reflection scans at 850 nm from the occlusal viewpoint (NIRR occlusal) and from trilateral evaluation (NIRR trilateral) as well as from digital bitewing radiography (BWR) and micro-computed tomography (µCT) using the Marthaler classification (score 0 to 4) and describing findings that were not assessable (na)

		BWR						NIRR occlusal		NIRR trilateral		
		0	1	2	3	4	na	0	1	0	1	Total
µCT	0	154	0	0	0	0	4	132	26	119	39	158
	1	19	0	0	0	0	0	14	5	12	7	19
	2	23	2	2	1	0	1	19	10	16	13	29
	3	24	4	7	6	0	1	27	15	19	23	42
	4	0	0	1	0	1	0	2	0	2	0	2
	Total	220	6	10	7	1	6	194	56	168	82	250

**Fig. 4:**
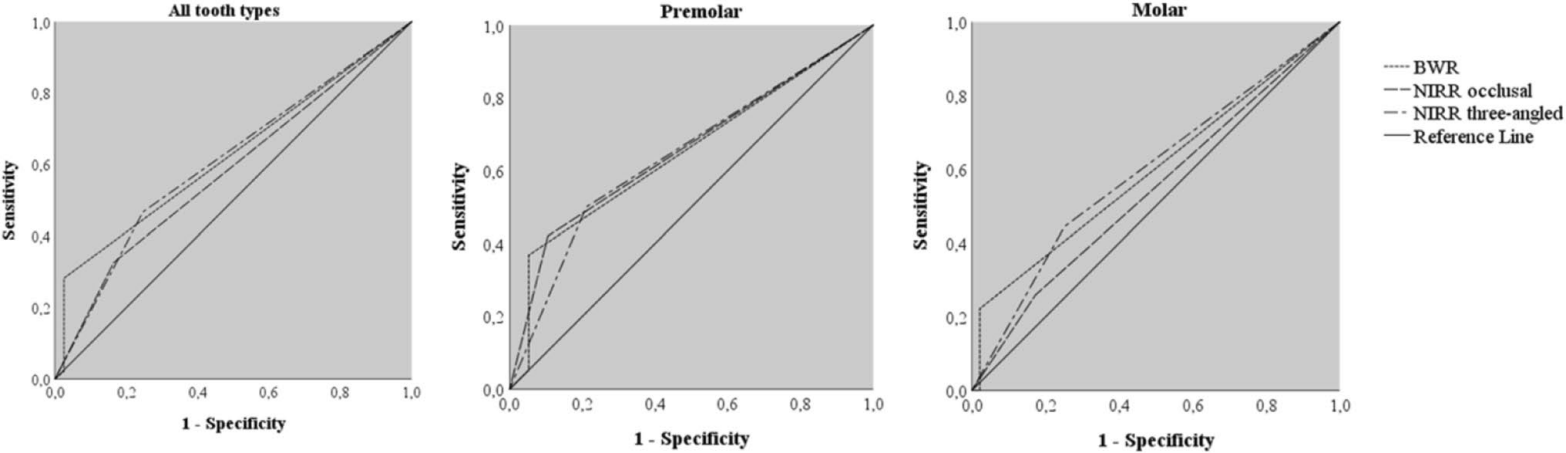
Receiver operating characteristic curves (ROCs) for carious lesions for all tooth types and separated into premolar and molar groups. The graphs show equal area under the ROCs for near-infrared reflection assessment from the occlusal viewpoint (NIRR occlusal), from three angles (NIRR trilateral) and for evaluation of bitewing radiography (BWR) (p < 0.05)

The unilateral NIRR imaging revealed an overall accuracy of 76.8%, an overestimation of 10.4% and an underestimation of 25.8%, while the trilateral assessment of NIRR resulted in an overall accuracy of 64.8%, an overestimation of 15.6% and an underestimation of 19.6%. For BWR, an overall accuracy of 71.2%, with no overestimation and an underestimation of 26.4%, was determined. In the latter analysis, 2.4% of all cases were not assessable due to overlap or anatomical artefacts. The inter- and intra-examiner reliability analysis (linear-weighted κ values) showed almost perfect or rather substantial agreement for NIRR and BWR (Table 2) [22]. High specificity values were found for NIRR and BWR, with slightly worse results for NIRR. When premolars and molars were analysed separately, the values for specificity remained consistently high. Low values (< 50%) for sensitivity were observed for all methods with slightly worse results for BWR. Occlusive NIRR achieved 6% higher sensitivity than BWR, and trilateral NIRR achieved 20% higher sensitivity than BWR. For premolars, the sensitivity values were slightly higher than for molars when assessed with NIRR from occlusal (Table 3). The sensitivity and specificity values are presented as ROC curves in Fig. 4. All AUC values for the different examination methods as well as further differentiation into tooth groups ranged between 0.5 and 0.7 and were classified as poor (Table 3). Multiple comparisons of AUC values revealed no significant difference between NIRR and BWR or within tooth type groups (p < 0.05). Using NIRR, the boundary of enamel and dentin was assessable in 118 (47.2%) samples. This was the case in 75.4% of the premolars and 38.9% of the molars (χ^2^ p < 0.001).

**Table 2:** Inter- and intra-examiner reliability (linear weighted κ values) for ratings of three-dimensional near-infrared reflection scans at 850 nm from the occlusal viewpoint (NIRR occlusal) and from trilateral evaluation (NIRR trilateral) as well as from digital bitewing radiography (BWR) with 0.95 confidence intervals (CI)

		Inter-examiner	Intra-examiner	
		Examiner 1 vs. Examiner 2	Examiner 1	Examiner 2
NIRR occlusal	κ	0.97	0.82	0.76
	Lower 0.95 CI	0.93	0.74	0.66
	Upper 0.95 CI	1.00	0.91	0.86
NIRR trilateral	κ	0.96	0.69	0.65
	Lower 0.95 CI	0.92	0.59	0.55
	Upper 0.95 CI	0.99	0.79	0.75
BWR	κ	0.85	0.90	0.91
	Lower 0.95 CI	0.76	0.85	0.85
	Upper 0.95 CI	0.93	0.96	0.97

**Table 3:** Sensitivity, specificity, false-positive (FP) value, false-negative (FN) value and area under the receiver operating characteristic curve (AUC) for evaluation of three-dimensional near-infrared reflection scans at 850 nm from the occlusal viewpoint (NIRR occlusal) and from trilateral evaluation (NIRR trilateral) and of digital bitewing radiography (BWR) using micro-computed tomography as a reference standard (lower and upper 0.95 confidence interval (CI) in parentheses)

		Sensitivity	Specificity	FP	FN	AUC
NIRR occlusal	All samples	0.33 (0.23–0.42)	0.84 (0.78–0.89)	0.16 (0.11–0.22)	0.67 (0.57–0.74)	0.58 (0.51–0.66)
	Premolars	0.42 (0.26–0.58)	0.89 (0.76–1.03)	0.11 (− 0.03–0.24)	0.58 (0.42–0.73)	0.66 (0.51–0.80)
	Molars	0.26 (0.14–0.38)	0.83 (0.76–0.89)	0.17 (0.11–0.24)	0.74 (0.62–0.81)	0.54 (0.45–0.64)
NIRR trilateral	All samples	0.47 (0.37–0.57)	0.75 (0.69–0.82)	0.25 (0.18–0.31)	0.53 (0.43–0.61)	0.61 (0.54–0.68)
	Premolars	0.50 (0.34–0.66)	0.79 (0.61–0.97)	0.21 (0.03–0.39)	0.50 (0.34–0.67)	0.65 (0.50–0.79)
	Molars	0.44 (0.31–0.58)	0.75 (0.68–0.82)	0.25 (0.18–0.32)	0.56 (0.42–0.64)	0.60 (0.51–0.69)
BWR	All samples	0.27 (0.17–0.36)	1.00 (1.00–1.00)	0.00 (0.00–0.00)	0.73 (0.64–0.80)	0.63 (0.55–0.70)
	Premolars	0.33 (0.18–0.49)	1.00 (1.00–1.00)	0.00 (0.00–0.00)	0.67 (0.51–0.81)	0.65 (0.50–0.80)
	Molars	0.22 (0.11–0.33)	1.00 (1.00–.100)	0.00 (0.00–0.00)	0.78 (0.67–0.84)	0.60 (0.50–0.69)

## Discussion

Our study investigated a 3D scanner that produces 2D images with NIR light in addition to real 3D data. Since the geometry between the optical elements used for the 3D data and 2D images is fixed, it is possible to map the 2D images onto digital 3D surface reconstructions using 3D range data (texture mapping).

To evaluate the diagnostic potential of the iTero Element 5D scanner, great care was taken to ensure an adequate study design. For example, the number of teeth to be examined was determined by estimating the number of cases, taking caries prevalence into account. For the sample size estimation, caries prevalence was determined to be 50%. This value may appear high at first glance when compared with the low DMFT values from epidemiological studies [23, 24]. Looking at the DMFT values across all age cohorts in Germany, the DMS V study showed a DMFT value of 11.2 in the 35–44 age cohort, and the DMFT value increased to 21.6 in the 75 + age group. However, it is important to note that these data only consider D3 and D4 lesions. The number of D1 or D2 lesions, which are a necessary prerequisite for the development of D3 and D4 lesions, is much higher, but there are hardly any population-representative studies for all age groups at the D1/D2 level. Recent studies in children found prevalence values on the order of over 70% in primary and mixed dentition [25, 26]. Because of the different systems of care, a conservative value of 50% for caries prevalence for initial defects seemed to be a good estimate for case number calculation. At this point, it is important to emphasize that we included only normally structured and restoration-free proximal surfaces of posterior teeth in this study. The reason was that we wanted to use the optical method primarily to detect initial defects whose progression could be delayed by non-invasive, preventive or microinvasive procedures, e.g., remineralization or infiltration [2, 27].

Until now, optical methods for caries diagnosis have often stored individual images in a database, and these images were used to uniquely identify the examined tooth. The combination of 3D data with diagnostic 2D information is novel. A significant advantage of this combination is that the examiners can easily orientate themselves in the process of diagnostic analysis by the tooth position in the dental arch and the tooth shape, which is not possible with database images. Especially with similar looking teeth, this is a simplification. This simplification became clear in our study, as we digitized only two teeth instead of an entire dental arch. Especially when the tooth shape was similar, orientation was not trivial on either the 3D datasets or the 2D images, so we had to mark the neighbouring tooth that was not evaluated with a dark stripe. However, as soon as the dental arch was available, this problem was eliminated, and the orientation corresponded to the information provided by the oral cavity.

However, this intuitive documentation is a minor aspect of the iTero Element 5D scanner in our research question. Numerous NIRR images acquired from different directions are saved at the same time as 3D measurements of the dental arch so that more information is available compared to the information provided by other NIRR scanners in which individual images are saved [9].

One of our objectives was therefore to assess whether the diagnostic performance of NIRR gains reliability if the teeth are evaluated from more than one preferred direction, for example, from the occlusal viewpoint but from all surfaces. In our study, we therefore performed caries diagnosis first from an occlusal viewpoint and second from trilateral images.

In our study, we found that decalcifications in enamel appeared perceptibly bright and almost radiant. Decalcification bands in the buccal or lingual region appeared very bright and rich in contrast. This could be due to the slightly longer wavelength of 850 nm. Alternatively, the brightness of decalcifications could be caused by simultaneous illumination of the tooth with a 680 nm laser diode used for the 3D scanner and with an 850 nm NIR LED. Red laser light could excite fluorescence in addition to illumination by pure reflection. Since the 1990s, diagnostic devices using laser fluorescence at a wavelength of 655 nm have been regarded as sensitive diagnostic tools for the detection of proximal caries [28]. The emitted light induces fluorescence of bacterial porphyrins, with intensity increasing proportionally to the depth of demineralization or bacterial contamination. However, in the absence of detailed information on the illumination strategy preferred by the manufacturer, this hypothesis cannot be tested.

Methodologically, it is also important to note the following details regarding BWR. For our study, all 250 specimens were radiographed once with and once without simulated proximal contact in a specially designed radiographic phantom to mimic the setup of the standard BWR (Fig. 3). Approximately 20% of the images acquired with proximal contact were not assessable due to overlap with adjacent teeth in the outer enamel region, reflecting a realistic clinical scenario [14]; thus, it can be inferred that our radiographic this reason, radiographic analysis was performed without simulated contact so that the maximum possible diagnostic information could be derived from the radiographs. Clinically, however, the results of the X-ray diagnostics would perform significantly worse than phantom generates clinically relevant data. However, if 20% of the findings were excluded due to superimposition, this would distort a comparison with optical methods. In our in vitro analysis, a significantly higher proportion of false-negative findings of BWR would have been expected, taking into account the overlaps of proximal contacts.

Another unique aspect of this study is the high number of 250 samples validated with µCT. Micro-computed tomography has become an attractive reference in diagnostic studies because a specimen can be reproducibly assessed in different sections and planes. The deepest part of a lesion, representing a 3D event with an irregular propagation pattern, can thus be determined and evaluated. In addition, the valuable sample pool is preserved and can be evaluated for further experiments.

Comparing both unilateral and trilateral NIRR results with BWR, the optical findings do not differ significantly from the radiography-based findings when AUC is chosen as the comparison criterion. If the proportions for FP and FN are also taken into account, it can be seen that there were no false-positive findings, but merely false-negative findings observed for BWR. This can be explained by two aspects. First, concerning the NIRR image at 850 nm, light scattering caused by carious lesions cannot be distinguished from light scattering caused by structural anomalies in the enamel. Second, enamel demineralization must be evident for caries to be clearly identified in a two-dimensional X-ray image [29]. Compared to X-ray images, optical methods are thus more sensitive to light scattering due to structural defects or even minimal demineralization defects. This has proven to be a general property of optical methods and is only confirmed here using the iTero Element 5D scanner as an example [30, 31]. Since these findings do not automatically lead to invasive therapeutic intervention, this sensitivity for proximal caries does not represent a disadvantage of the methods. If these findings sensitize the patient and motivate him to engage in preventive behaviour, this scan will provide a desirable result, the success of which can even be controlled in the long term through repeated monitoring at any time.

When observing the values of the inter- and intra-examiner reliability assessment, it is noticeable that inter-examiner agreement showed higher results than the corresponding data of the re-testing procedure for the intra-examiner reliability test. The results of the intra-examiner assessment decreased slightly from the first test to the re-test. This can possibly be interpreted as a result of the fact that the questionable findings were discussed between the examiners after the first test, as decalcification bands and reflection artefacts caused difficulties in the assessment of the surfaces. This led to the substantial outcome of the intra-examiner reliability assessment of NIRR, which was lower for the trilateral assessment, as these results are composed of three values for occlusal, buccal and lingual. It can be reasoned that the resulting in-between calibration of the examiners biased the results. A consensus discussion for both cycles at the end of the second evaluation cycle would probably have led to more objective results. It is likely that the latter variant would have resulted in lower inter-examiner agreement.

Differentiation of enamel and dentin tissue by NIRR was successful in 47.2% of samples, more so for premolars than molars. This low potential for discrimination of dental hard tissues at 850 nm of NIRR has been reported previously [9, 32]. One reason for the unequal detection of the enamel-dentin interface in premolars and molars could be the difference in enamel thickness or the radius of the proximal contact area, which is usually smaller in premolars as contrast decreases with increasing enamel thickness [30]. Due to the better visibility of the EDJ in premolars, we additionally performed a statistical analysis of the values SE, SP, FP, FN and AUC specifically for molars and premolars where the EDJ was visible. No significant differences were found, except for the proportion between the occlusal view of molars with invisible EDJ and premolars with visible EDJ. Since the EDJ was identifiable in less than half of the surfaces, this can be considered a minor aspect of the results.

Due to the difficult visibility of the EDJ with the iTero Element 5D scanner, in contrast to the evaluation of BWR and µCT data, there was no assessment of whether the EDJ was crossed. Consequently, NIRR findings were evaluated only in terms of a dichotomous decision: healthy or diseased. While NIRR can detect initial defects in the enamel with high sensitivity, it cannot, in contrast to BWR, support a reliable recommendation for or against invasive therapy when the enamel/dentin boundary is exceeded. With NIRR, a more detailed classification of lesion severity may be possible in higher NIR ranges of approximately 1300 nm, as enamel becomes more transparent and shows less scattering and absorption with increasing wavelengths [33–35]. However, such longer wavelength systems require a special indium-gallium-arsenide camera sensor, as the silicon sensors used have sufficient efficiency only up to approximately 850 nm. These special sensors are currently still very expensive and therefore do not seem to be an option for commercially available NIR caries detection devices at present [36].

The differences between the unilateral and trilateral findings are compared in detail in Table 3. Based on the area under the receiver operating characteristic curve, despite minor differences in sensitivity, specificity, FP and FN, it can be summarized that no substantial difference was observed between unilateral and trilateral imaging. It was to be expected, however, that trilateral imaging would provide additional information in the area of the proximal surfaces. One explanation for this could be that image data were often missing, especially in the area of the proximal contacts. This might be due to the shielding of NIR illumination. The NIR light source can only be mounted next to the video sensor, so the light source cannot achieve exact coaxial illumination. Due to the narrow access to proximal contacts, important information is therefore missing, especially in the region of interest. This problem can probably be solved by positioning several light sources in a circular fashion around the video sensor. This could be difficult due to technical limitations but should be considered an option to increase the diagnostic potential of future sensor generations.

**Fig. 5:**
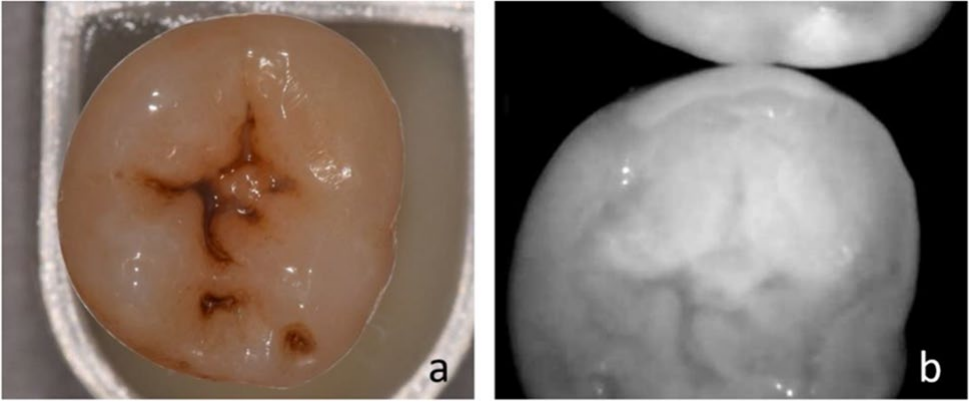
The molar (**a**) reveals the white border artefacts in the area of the marginal ridge (**b**) which are probably created due to the spherical shape of the proximal surface

In the NIRR image from the occlusal viewpoint, proximal caries can only be reliably diagnosed up to a certain distance from the occlusal surface, as long as the caries lesion is not too far below a non-carious proximal contact point (Fig. 3). As soon as the caries lesion is localized deep below the contact point, detection with only one occlusal image reaches its limits. Here, additional lateral images may be essential for diagnosis. Lederer et al. found that the distance of a proximal lesion from the occlusal surface significantly influenced its visibility [9]. The results presented in Table 1 confirm this fact, as the two deeper dentin lesions included in the sample pool were not detected at all by the new method. These were located below the proximal contact but still above the enamel-cement border. However, in this case, the trilateral view also failed to detect the lesion. It must be mentioned that there are too few samples with advanced dentin lesions in the sample pool to make a significant statement at this point. In addition, artefacts also hampered the performance of the scanner. In the proximal region, white marginal artefacts were observed in the area of the marginal ridge (Fig. 5) [9]. Consequently, underlying incipient caries could not be detected in all these cases, while larger lesions were easier to identify.

**Fig. 6:**
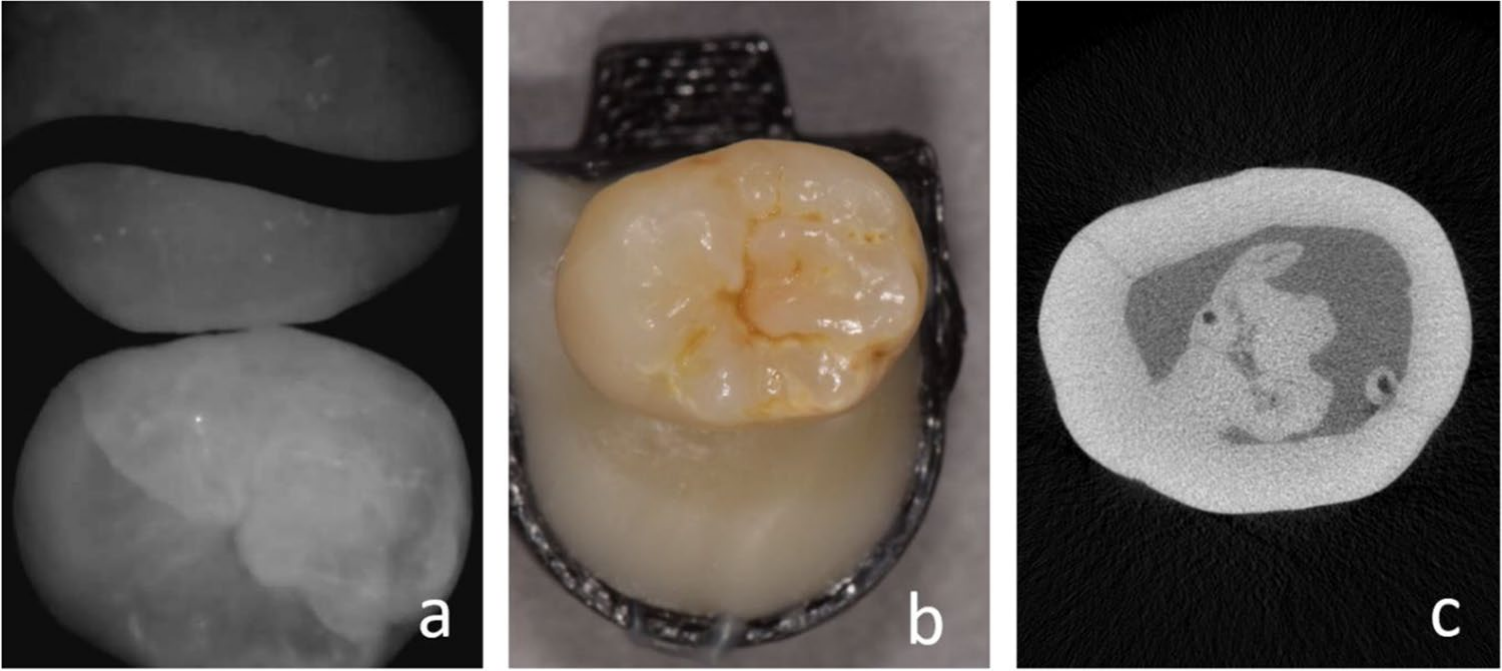
The diagonal crack extending through the clinical crown of a premolar cannot be identified with white light (***b***) but can be detected by near-infrared reflection (***a***) and micro-computed tomography (***c***)

Coincidentally, tooth cracks were detectable by NIRR assessment, which was not possible under white light (Fig. 6). The diagnosis of enamel cracks would be a benefit of 3D NIR imaging, but since enamel cracks do not result in a therapeutic consequence, this observation is of secondary clinical importance. Unlike other NIRR devices for caries diagnosis that use 850 nm LEDs as a light source, the iTero Element 5D scanner does not show any reflection artefacts caused by a smooth dental surface. Images acquired from the NIRR scanner present light scattered in depth mainly at dentin and irregularities in enamel, without being superimposed by superficial specular reflections, as has been observed for other NIRR diagnostic devices [9].

The novel approach to entirely measure the dental arch from different directions can be an attractive option for the development of future diagnostic applications. It would be possible to calculate the complete surface texture for the entire 3D data set from the multitude of individual images. One approach, for example, would be to use more than one image at a time for this purpose and to eliminate the image noise by averaging several images. However, no information is available from the manufacturer on these options, so this aspect is currently not assessable.

## Conclusions

In this study, a method for the investigation of optical diagnostic systems was developed, which ensures both quantitative and objective caries diagnosis based on a µCT reference. Based on the AUC, NIRR diagnostics of the iTero Element 5D scanner achieved diagnostic results comparable to those of BWR*.* NIRR with and without the trilateral information can detect initial defects in the enamel with higher sensitivity than BWR, but it cannot, in contrast to BWR, support a reliable recommendation for or against invasive therapy when the EDJ is exceeded.
